# Effects of Dietary Folic Acid Supplementation on Productive Performance, Egg Quality, Flavor, and Folate Species Deposition in Laying Hens

**DOI:** 10.3390/ani16121756

**Published:** 2026-06-06

**Authors:** Wen Li, Junhong Miao, Zhenxu Li, Shuhui Yan, Mengfei Zhu, Kaidong Liu, Kai Zhang

**Affiliations:** 1 College of Animal Science and Technology, Qingdao Agricultural University, Qingdao 266109, China; 20242109058@stu.qau.edu.cn (W.L.); junhong-miao@stu.qau.edu.cn (J.M.); 20242109019@stu.qau.edu.cn (Z.L.); 20232209016@stu.qau.edu.cn (S.Y.); 20252109043@stu.qau.edu.cn (M.Z.); 2 Qingdao Institute of Animal Science and Veterinary Medicine, Qingdao 266199, China

**Keywords:** folic acid, productive performance, egg quality, laying hens, egg yolk folate species

## Abstract

This study explored the effects of varying levels of dietary folic acid (FA) supplementation on laying performance, egg quality, egg yolk flavor, and the deposition of four folate forms in eggs. A total of 336 Hy-Line Brown layers were randomly divided into seven groups and fed basal diets supplemented with FA at doses of 0, 0.25, 0.5, 1, 5, 10 and 15 mg/kg, respectively. The results suggested that dietary FA supplementation exerted no notable effects on laying performance or egg quality. Electronic nose analysis of egg yolk flavor revealed that high FA supplementation levels (10 and 15 mg/kg) linearly decreased sensor response scores to organic sulfides, aroma constituents, alkanes, and alkane aromatic components compared to the low-dose FA groups (0 to 0.5 mg/kg). Analysis of egg yolk folates identified four folate forms with the following proportions: 5-methyltetrahydrofolate (5-MTHF, 79–84%), 10-formylfolic acid (10-FMF, approximately 10%), folic acid (<5%), and 5-formyltetrahydrofolic acid (5-FMTHF, <3%). Linear and quadratic relationships were observed between dietary FA supplementation levels and the content of three folate forms (5-MTHF, folic acid, and 10-FMF) in egg yolk and whole eggs. Notably, the egg highest total folate content (119.60 μg/100 g) was observed in hens fed diets containing 15 mg/kg FA at week 4, although this was not significantly different from hens fed 5 mg/kg FA at week 6. In summary, 5 mg/kg FA was the optimal dose to achieve saturated total folate content in eggs without altering egg flavor.

## Simple Summary

Folate deficiency is recognized as a global issue, with more than 4 billion people—approximately 54% of the world’s population—not consuming sufficient folate. Folic acid-fortified eggs serve as an excellent natural dietary source of highly bioavailable folate. However, aside from 5-methyltetrahydrofolate, knowledge regarding the deposition of other folate forms (e.g., synthetic folic acid) in eggs in response to dietary folic acid supplementation remains limited. Furthermore, whether dietary folic acid supplementation affects egg flavor requires further investigation. Therefore, the present study aimed to examine the effects of folic acid supplementation on laying performance, egg quality, flavor, and folate species deposition in laying hens.

## 1. Introduction

Folate, a water-soluble B vitamin, is a generic term encompassing pteroylglutamic acid and related compounds exhibiting similar biological activity. It serves as an important donor and carrier of one-carbon transfer reactions, supporting DNA synthesis and amino acid metabolism [1]. For women, adequate folate intake before conception and during early pregnancy is essential to prevent neural tube defects in infants [2]. Meanwhile, folate deficiency has been demonstrated to be correlated with various health problems, including macrocytic anemia, cardiovascular disease, and neurodegenerative disorders [3]. The U.S. National Institutes of Health recommends a daily folate intake of 400–600 μg for adults (including pregnant and lactating women), with a tolerable upper limit of 1000 μg/day. However, folate deficiency is recognized as a global problem, and more than 4 billion people (54% of the global population) do not consume enough folate [4].

Eggs are an excellent natural dietary source of highly bioavailable folate [5]. Previous studies have confirmed that dietary supplementation with synthetic folic acid (FA) can markedly elevate the folate content in eggs [6,7,8,9,10,11,12]. However, published data indicate that findings regarding the folate content in eggs are inconsistent. Furthermore, previous studies have primarily focused on the enrichment of total folate or 5-methyltetrahydrofolate (5-MTHF) in eggs [6,10,11,13]. Aside from 5-MTHF, 5-formyltetrahydrofolic acid (5-FMTHF), 10-formylfolic acid (10-FMF), and FA were also found in egg yolks [8,14]. As the active form of folate, 5-MTHF is an ideal supplement, especially for the nearly 40% of the global population who carry MTHF reductase polymorphisms [15]. It is worth noting that long-term, high-dose consumption of unmetabolized FA (synthetic FA) may pose health risks, such as masking the symptoms of vitamin B_12_ deficiency and accumulation of homocysteine [16,17,18]. Although 5-MTHF is considered the primary form of folate deposited in egg yolk [19,20], data on unmetabolized folic acid (FA) and other folate forms in response to dietary FA supplementation remain limited, underscoring the need for further research.

Moreover, as consumers increasingly seek high-quality eggs, flavor, in particular, has garnered greater attention. Recently, Yu et al. (2024) reported that a high dose of FA supplementation reduced the fatty acid and amino acid profiles in fortified eggs [11]. Therefore, when evaluating the quality of folate-enriched eggs, only detecting the folate content is not sufficient, and the egg flavor should also be considered. Previous studies have evaluated egg quality traits (e.g., eggshell quality, Haugh unit, and albumen height) in response to dietary FA supplementation [9,10]; however, few reports have examined the effects of FA supplementation levels on egg flavor. Traditionally, flavor evaluation relied primarily on subjective assessment, and the results were often influenced by several factors. Electronic nose technology, which employs artificial sensor arrays to mimic the human sense of smell, provides a more accurate method for evaluating egg flavor by overcoming the limitations of conventional analytical techniques [21,22].

Therefore, this research aimed to explore the effects of dietary FA on laying performance, egg quality, flavor (evaluated using an electronic nose), and the content of four forms of folate in the egg yolk of laying hens. These findings will provide novel insights for the production of folate-fortified eggs.

## 2. Materials and Methods

### 2.1. Chemicals and Reagents

The analytical standard of FA (CAS No. 59-30-3) was obtained from Sigma-Aldrich (St. Louis, MO, USA). 5-FMTHF (CAS No. 58-05-9), 5-MTHF (CAS No. 31690-09-2), and 10-FMF (CAS No. 134-05-4) were obtained from Shanghai ZZBIO Co., Ltd. (Shanghai, China).

### 2.2. Ethical Approval

All experimental protocols involving animals were approved by the Animal Care and Use Committee of Qingdao Agricultural University (DKY2025023).

### 2.3. Animals, Diets, and Sample Collection

A total of 336 Hy-Line brown laying hens (40-week-old) were randomly assigned to seven groups, with six replicates per group and eight hens per replicate (reared together in one cage). The seven groups received a basal diet (Table 1) supplemented with 0, 0.25, 0.5, 1, 5, 10, and 15 mg/kg of synthetic FA (CAS No. 59-30-3), respectively. The NRC (1994) reports that the folate requirement for laying hens is 0.25 mg/kg of diet [23]. The experiment lasted for 12 weeks. The analyzed folate content of the basal diet was 0.29 mg/kg, derived solely from feed ingredients, with no exogenous folate supplementation. All hens were purchased from Qingdao Jimo Birdsong Farming, a local commercial farm. Hens were raised in battery cages (450 cm^2^ per bird) in a controlled environment and kept under a 16 h photoperiod. All laying hens were provided free access to feed and water. Eggs were collected and weighed daily to calculate the average egg weight (AEW) and laying rate (calculated as the ratio of the number of eggs collected to the number of birds), respectively. Feed intake was recorded during the 4th, 8th, and 12th weeks and used to calculate the average daily feed intake (ADFI) and feed-to-egg ratio (F:E).

### 2.4. Egg Quality Analysis

Three eggs per replicate were collected at weeks 6 and 12 of the experiment for egg quality evaluation. Eggshell strength was measured using the EFG-0503 tester (Robotmation, Tokyo, Japan), while yolk color, albumen height, and Haugh unit were assessed with the EMT-5200 integrated egg quality analyzer (Robotmation, Tokyo, Japan).

Sensory analysis of egg yolk using an electronic nose was conducted as previously [24]. In brief, approximately 1.0 g of egg yolk sample was transferred into a 10 mL headspace vial. The sample was maintained at 20 °C for 20 min, followed by incubation in a water bath at 80 °C for 20 min. After cooling naturally for 30 min, the volatile flavor compounds of the egg yolk were analyzed using the PEN3 electronic nose system (AIRSENSE Analytics GmbH, Schwerin, Germany).

### 2.5. Egg Yolk Folate Speciation Analysis Using HPLC-MS/MS

At the 4th, 6th, and 12th weeks of the experiment, one egg from each replicate was collected and weighed. The egg yolk was then separated, weighed, and stored at −20 °C. The concentrations of FA, 5-MTHF, 5-FMTHF, and 10-FMF in the egg yolk were analyzed following a previous report with minor modifications [25]. Briefly, approximately 0.3 g of freeze-dried egg yolk powder was mixed with 3 mL of 50 mM phosphate buffer (containing 1% sodium L-ascorbate and 0.1% β-mercaptoethanol, pH 7.4), vortexed for 6 min, and then subjected to ultrasound for 5 min. Next, 7 mL of methanol was added, followed by vortexing for 6 min and centrifugation at 8000 *g* for 10 min. Three milliliters of the supernatant were passed through an ERM-Lipid column (Agilent Technologies, Santa Clara, CA, USA) and filtered using 0.22 μm filters. Subsequently, 200 μL of the resulting filtrate was mixed with 800 μL of ultrapure water for HPLC-MS/MS analysis, as described previously [25].

### 2.6. Statistical Analysis

Statistical analysis was performed using one-way ANOVA with RStudio (RStudio/2026.01.2+418). Duncan’s multiple range test was used for multiple comparisons to assess differences between groups. Linear and quadratic trends in response to dietary FA supplementation were further assessed using orthogonal polynomial contrasts. All data are expressed as mean ± standard error, with *p* < 0.05 considered statistically significant. GraphPad Prism 10.0 was used to create the experimental figures (GraphPad Software Inc., La Jolla, CA, USA).

## 3. Results

### 3.1. Productive Performance and Egg Quality

As shown in Table 2, dietary FA supplementation has no obvious effect on the laying rate, ADFI, AEW, and F:E ratio in laying hens. However, during weeks 5 to 8 of the experiment, hens fed diets supplemented with 1 to 10 mg/kg FA tended to have a higher laying rate compared with the 0.5 mg/kg FA treatment (*p* = 0.056). According to Table 3, the egg quality traits (e.g., Haugh units, eggshell strength, and yolk color) were not significantly affected by dietary FA supplementation.

### 3.2. Egg Yolk Flavor

**Figure 1:**
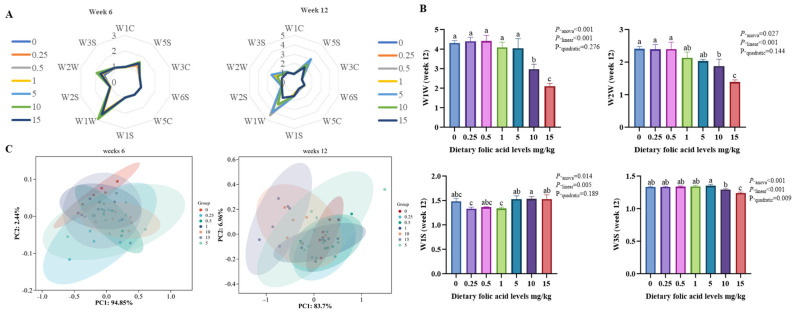
Effects of dietary supplementation with various levels of FA on yolk flavor score (**A**,**B**) and PCA score plots (**C**). Different lowercase letters of peer shoulder notes indicate significant differences (*p* < 0.05).

The flavor scores of eggs in response to dietary FA supplementation were evaluated using an electronic nose (Figure 1A). Compared with the 0 and 0.25 mg/kg FA groups, the 10 and 15 mg/kg FA groups exhibited linear decreases in sensor responses of W1W (organic sulfides), W2W (aromatic compounds/organic sulfides), and W3S (alkanes) (*p* < 0.05; Figure 1B). Conversely, the W1S response (methane) was higher in the 5–15 mg/kg FA groups than in the 0.25 mg/kg group (*p* < 0.05), indicating a significant linear dose-dependent effect of FA supplementation. PCA revealed that egg flavor scores of the 10 and 15 mg/kg FA groups were distinctly separated from those of the 0 and 0.25 mg/kg groups (Figure 1C).

### 3.3. 5-MTHF Content in Eggs

According to Table 4, the 5-MTHF content in eggs was expressed in μg per 100 g of dried egg yolk and per 100 g of fresh egg. Compared to the 0 mg/kg FA group, egg yolk and whole egg 5-MTHF content increased both linearly and quadratically with higher FA supplementation during weeks 4, 6, and 12 (*p* < 0.001). For instance, a linear and quadratic dose–response relationship was observed between the 5-MTHF content in dried egg yolk and dietary FA supplementation levels (0, 0.5, 1, and 10 mg/kg) in hens after 4 weeks of the experiment (*p* < 0.001). At 6 weeks, the 10 mg/kg FA group exhibited a peak 5-MTHF content of 572.19 ± 26.75 μg/100 g of dried egg yolk; however, there was no significant difference compared to hens fed 15 mg/kg FA. At 12 weeks, the highest dried egg yolk 5-MTHF content (511.73–530.76 μg/100 g) was observed in hens fed diets containing 5 to 15 mg/kg FA, which was significantly higher (*p* < 0.001) than that in the 1 mg/kg FA group (437.74 ± 10.78 μg/100 g of dried egg yolk). Meanwhile, no significant difference was found in the 5-MTHF content in dried egg yolk among hens fed 0 to 0.5 mg/kg FA diets at 12 weeks of the experiment.

### 3.4. FA Content in Eggs

In Table 5, compared to the 0 mg/kg FA group, the FA (unmetabolized FA) content in egg yolk and whole eggs increased linearly with higher FA supplementation during week 4 (*p* < 0.001). Additionally, both linear and quadratic effects were observed for FA deposition during weeks 6 and 12 (*p* < 0.05). The highest folic acid content (29.93 μg/100 g of dried yolk or 5.23 μg/100 g of egg) was observed in hens fed diets containing 15 mg/kg FA at week 4 of the experiment.

### 3.5. 5-FMTHF Content in Eggs

As shown in Table 6, dietary FA supplementation levels did not significantly interact with 5-FMTHF content in egg yolk or whole eggs during weeks 4 and 6. Interestingly, the 5-FMTHF content in egg yolk (11.86 μg/100 g) and whole eggs (2.08 μg/100 g) from hens fed a diet containing 0.5 mg/kg FA was significantly higher than that in hens fed diets containing 0, 1, and 5 mg/kg FA (*p* < 0.01) during week 12 of the experiment.

### 3.6. 10-FMF Content in Eggs

During week 4 of the experiment, increased dietary FA supplementation produced both linear and quadratic effects on 10-FMF content in egg yolk and whole eggs (*p* < 0.05; Table 7). However, during weeks 6 and 12, the 10-FMF content in egg yolk and whole eggs increased linearly with higher dietary FA supplementation (*p* < 0.01). At week 4, the 10-FMF content increased from 38.44 μg/100 g of dried yolk in the 0 mg/kg FA group to the highest level of 76.21 μg/100 g of dried yolk in hens fed 15 mg/kg FA (*p* < 0.001), with no significant difference observed between the 10 mg/kg and 15 mg/kg FA groups. Similar patterns of 10-FMF enrichment were observed at weeks 6 and 12 of the experiment in response to dietary FA supplementation.

### 3.7. Total Folate Content and the Percentage of Folate Species in Eggs

The total folate content in eggs was calculated based on four folate species (5-MTHF, FA, 5-FMTHF, and 10-FMF) detected in the egg yolks. As shown in Table 8, the total folate content in egg yolk and whole eggs increased both linearly and quadratically in response to dietary FA supplementation during weeks 4, 6, and 12 of the experiment (*p* < 0.001), exhibiting enrichment patterns similar to those of 5-MTFH in the egg yolk and whole eggs. At week 4, the maximum total folate content (685.31 μg/100 g dried egg yolk or 119.61 μg/100 g whole egg) was observed in hens fed 15 mg/kg of FA. By week 12, total folate accumulation stabilized within a saturation range of 605.03–635.25 μg/100 g dried yolk and 106.50–112.71 μg/100 g whole egg in the groups receiving 5–15 mg/kg FA.

**Figure 2:**
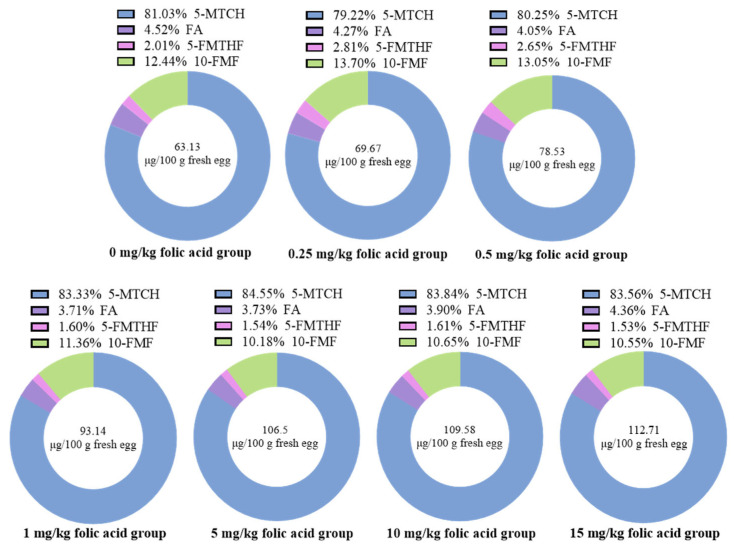
Effects of dietary supplementation with various levels of FA on the percentage of folate species in whole eggs.

Figure 2 presents the percentages of four folate species in eggs for all groups at week 12 of the experiment. 5-MTHF is the primary form of folate accumulated in eggs, accounting for 79.2% to 84.6% of the total FA per 100 g of eggs. Second, 10-FMF accounts for 10.2% to 13.7% of the total FA; third, FA accounts for 3.7% to 4.5%; and finally, 5-FMTHF accounts for the lowest proportion of 1.5% to 2.8%.

## 4. Discussion

Laying performance and egg quality traits (e.g., eggshell strength, albumen height, and Haugh unit) directly influence economic benefits and consumers’ purchasing decisions [26]. In the present study, FA supplementation had no significant effects on laying rate, AEW, ADFI, F:E, and egg quality parameters such as eggshell strength, yolk color, and Haugh unit in laying hens. These findings are consistent with results reported in previous studies [7,11,27]. Findings from Bagheri et al. indicated that FA supplementation at 2–15 mg/kg improved laying rate and egg shape index while reducing the F:E ratio in aged laying hens [10]. These results indicate that FA supplementation positively affects productive performance and egg quality in late-laying hens, but not during the peak laying period. Notably, no significant negative effects were observed in hens on productive performance and egg quality fed 0 mg/kg FA in our study, which may be due to sufficient FA reserves in the hens’ tissues from pretrial diets or feed ingredients, adequately supporting production needs.

Egg flavor is a significant indicator of quality for the comprehensive evaluation of fortified eggs. However, evaluating egg flavor is a more challenging task than analyzing routine egg quality parameters, such as Haugh units and yolk color. The electronic nose is a novel bionic device that mimics biological olfaction to detect and characterize various gases and odors, featuring low cost, short response time, and rapid detection [28]. Recently, electronic nose technology has been employed as an alternative method for human biological monitoring in evaluating egg flavor and distinguishing eggs from different breeds of laying hens [29,30,31]. To our knowledge, no studies have evaluated the effects of FA supplementation levels on egg flavor. In the present study, we successfully evaluated changes in egg yolk flavor in response to dietary FA supplementation levels ranging from 0 to 15 mg/kg. The PCA analysis successfully separated the egg yolk flavor scores between the high FA supplementation groups and the low FA supplementation groups. Similarly, Wang et al. reported that an electronic nose could distinguish eggs stored under cool and room-temperature conditions using PCA analysis [32]. Additionally, our results indicated that high levels of FA supplementation (10 and 15 mg/kg) decreased flavor scores related to alkane aromatic components, organic sulfides, and alkanes, while flavors scores associated with methane increased. Lipids are the primary components of egg yolks and serve as important precursors in the development of their flavor [33]. Interestingly, Yu et al. found that dietary supplementation with 15 mg/kg FA significantly decreased the levels of monounsaturated fatty acids and amino acids (e.g., aspartic acid and glutamic acid) in eggs compared to lower FA levels (5 and 10 mg/kg) [11]. Therefore, we speculate that the effect of high FA supplementation on the flavor of egg yolks may be due to its influence on the lipid composition of the yolks. However, the observed changes in volatile odor profiles cannot yet be directly correlated with specific sensory flavor attributes. Furthermore, it remains unclear whether high-dose folic acid supplementation enhances or diminishes the overall flavor of egg yolk.

Prior research has revealed that 95% of the folate in eggs is found in the egg yolk [13]. Strandler et al. reported that no folates were detected in egg whites [34]. Therefore, we chose to measure the folate content in egg yolks. In our present study, 5-MTHF was the major folate form deposited in egg yolk, accounting for >80% of the total folates, followed by 10-FMF (approximately 10%), FA (<5%), and 5-FMTHF (<3%). Except for FA, our present findings are consistent with the findings of Sun et al. that four folates were detected in egg yolk based on the HPLC-MS/MS method [25]. But Sun et al. found that the proportion of FA in eggs was less than 2% of the total folate, whereas in our study, this proportion ranged from 3.7% to 4.5%, which is consistent with the results (2.3–5.1%) reported by Hoey et al. [8]. These results indicate that laying hens are highly efficient at converting dietary synthetic FA into active folate (5-MTHF) for deposition in eggs. However, previous studies have reported that except for 5-MTHF, only 10-FMF and FA were found in egg yolk using HPLC and microbiological assay approaches [8,34,35]. This discrepancy may be attributed to differences in analytical methods and the pre-treatment protocols of folate extraction in egg yolk [36].

According to our results, linear and quadratic effects were observed between dietary FA supplementation levels and 5-MTHF deposition in egg yolk and whole eggs, consistent with previous reports [6,8,9]. In our study, the highest content of 5-MTHF deposition in eggs (56.49 μg/egg, 100.29 μg/100 g of whole egg, or 572.19 μg/100 g of dried egg yolk) was observed in hens fed diets containing 10 mg/kg FA at week 6 of the experiment. The obtained data are consistent with previous observations reported by Bagheri et al. (54.5 μg/egg) [10], Dickson et al. (57.9 μg/egg) [9], and Sun et al. (599 μg/100 g of egg yolk) [25]. In contrast, Zhao et al. reported a maximum 5-MTHF content of 120 μg/egg when feeding diets containing 4 mg/kg folic acid for 16 weeks [12]. Interestingly, we found that the dosage required to saturate total folate levels in eggs gradually decreased over time. The plasma serves as the precursor pool for yolk folate deposition. Circulating plasma folate concentrations reach a saturated state under high dietary FA supplementation [13], which partially explains why egg folate levels plateau. Furthermore, the optimal supplemental level for achieving the maximum total folate content in eggs was found to range between 4 and 128 mg/kg [6,7,8,13]. These discrepancies may be attributed to differences in breeds (commercial laying hens vs. broiler breeders), variability in feed intake, and variations in egg weight. Previous findings from House et al. and Hebert et al. revealed that differences in basal dietary folate (endogenous folate) levels (0.49 mg/kg vs. 1.45 mg/kg) had little effect on egg folate deposition (17.5 μg/egg vs. 16.7 μg/egg) [6,7]. Similar findings can also be confirmed by comparing the results of the present study with those of Hoey et al. [8]. Significantly, the type of dietary cereal influenced the folate content deposited in eggs, which may be attributed to the adverse effects of non-starch polysaccharides [37]. Compared with a wheat-based diet, hens fed a corn-based diet showed higher folate deposition in their eggs. These results suggest that optimal dietary FA levels for producing folate-fortified eggs vary with dietary cereal types. Notably, unmetabolized FA concentrations in egg yolk were linearly correlated with dietary FA supplementation levels. However, the maximum content of unmetabolized FA in eggs reached only 5.23 μg per 100 g egg, which was well below the 400 μg/d threshold at which unmetabolized FA began to enter human blood circulation [38]. Therefore, folate-fortified eggs are a highly safe and effective dietary source of folate.

## 5. Conclusions

The results of this study demonstrated that dietary FA supplementation had no significant impact on laying performance or egg quality in laying hens. However, the egg yolk flavor was altered by high doses of FA supplementation (10 and 15 mg/kg). Four folate forms were detected in egg yolk, distributed as follows: 5-MTHF (>80%), 10-FMF (approximately 10%), folic acid (<5%), and 5-FMTHF (<3%). Linear and quadratic effects were observed between the levels of 5-MTHF, 10-FMF, and FA in egg yolk and whole eggs and the levels of dietary FA supplementation. Overall, total folate saturation was reached in hens fed diets containing 5 to 15 mg/kg FA by week 12 of the experiment, suggesting that a diet with 5 mg/kg FA may be optimal for producing folate-fortified eggs.

## Figures and Tables

**Table 1:** Composition and nutrient levels of the basal diet.

Ingredients	Content
Corn, %	62.5
Soybean meal, %	25.0
Soybean oil, %	1.0
Limestone, %	8.5
Premix ^1^	3
Total	100
Nutrient levels ^2^	
ME, kcal/kg	2710
CP, %	16.04
Ca, %	3.50
Available P, %	0.36
Lys, %	0.82
Met, %	0.38
Thr, %	0.61
Try, %	0.19
Folate, mg/kg	0.29

Note: ^1^ The premix provided the following per kg of diets: Vitamin A (trans-retinyl acetate) 20,000 IU; Vitamin D_3_ (cholecalciferol) 2500 IU; Vitamin E (all-rac-a-tocopherol acetate) 15 IU; Vitamin K_3_ 0.75 mg; Vitamin B_1_ (thiamin) 1.5 mg; Vitamin B_2_ (riboflavin) 3.8 mg; Vitamin B_6_ (pyridoxine HCl) 4.5 mg; Vitamin B_12_ (cobalamin) 0.01 mg; biotin 0.15 mg; folic acid 0 mg; D-pantothenic acid 10 mg; nicotinic acid 30 mg; choline (as choline chloride) 500 mg; Cu (as copper sulfate) 10 mg; Fe (as ferrous sulfate) 80 mg; Mn (as manganese sulfate) 80 mg; Zn (as zinc sulfate) 90 mg; I (as potassium iodide) 0.40 mg; Se (as sodium selenite) 0.30 mg; Met (as dl-Met) 0.12%; available P (as CaHPO_4_) 0.36%; NaCl 0.20%. ^2^ The nutrient levels were calculated values (except for CP and folate).

**Table 2:** Effects of dietary supplementation with various levels of FA on productive performance of laying hens.

Item	Laying Rate, %	ADFI, g	AEW, g	F:E
Weeks 1 to 4				
0 mg/kg FA	91.67	116.64	62.35	2.04
0.25 mg/kg FA	92.50	116.47	62.36	2.02
0.5 mg/kg FA	88.67	117.56	63.20	2.10
1 mg/kg FA	93.00	118.49	62.66	2.04
5 mg/kg FA	91.83	118.11	62.06	2.08
10 mg/kg FA	92.17	115.07	61.66	2.03
15 mg/kg FA	90.83	116.25	61.98	2.07
SEM	0.51	0.34	0.19	0.01
*P*-ANOVA	0.362	0.093	0.427	0.623
*P*-Linear	0.917	0.101	0.085	0.794
*P*-Quadratic	0.474	0.829	0.372	0.940
Weeks 5 to 8				
0 mg/kg FA	92.17	123.31	63.93	2.10
0.25 mg/kg FA	94.17	123.35	63.70	2.06
0.5 mg/kg FA	89.67	124.31	64.68	2.15
1 mg/kg FA	95.00	122.72	64.01	2.02
5 mg/kg FA	95.67	123.59	64.63	2.03
10 mg/kg FA	95.67	122.60	63.64	2.01
15 mg/kg FA	91.17	122.39	63.98	2.10
SEM	0.65	0.29	0.18	0.01
*P*-ANOVA	0.056	0.627	0.750	0.125
*P*-Linear	0.939	0.165	0.606	0.694
*P*-Quadratic	0.011	0.831	0.398	0.035
Weeks 9 to 12				
0 mg/kg FA	92.00	125.94	64.81	2.12
0.25 mg/kg FA	92.00	128.60	64.09	2.18
0.5 mg/kg FA	91.67	128.32	65.42	2.14
1 mg/kg FA	95.67	127.97	64.72	2.07
5 mg/kg FA	94.67	126.91	64.41	2.08
10 mg/kg FA	93.67	126.49	64.52	2.10
15 mg/kg FA	92.00	126.74	65.09	2.12
SEM	0.64	0.34	0.17	0.02
*P*-ANOVA	0.538	0.281	0.467	0.608
*P*-Linear	0.959	0.219	0.756	0.680
*P*-Quadratic	0.158	0.678	0.307	0.308

**Table 3:** Effects of dietary supplementation with various levels of FA on egg quality of laying hens.

Item	Egg Shell Strength, kg·cm^−2^	Albumen Height, mm	Haugh Unit	Yolk Color
Weeks 6				
0 mg/kg FA	3.92	7.72	87.46	5.88
0.25 mg/kg FA	3.77	8.29	89.87	6.00
0.5 mg/kg FA	3.76	7.77	86.43	5.83
1 mg/kg FA	3.81	8.08	88.97	6.02
5 mg/kg FA	3.71	7.99	88.19	6.13
10 mg/kg FA	4.00	8.46	90.69	5.45
15 mg/kg FA	3.93	8.57	91.36	6.38
SEM	0.69	0.11	0.63	0.12
*P*-ANOVA	0.926	0.327	0.361	0.622
*P*-Linear	0.427	0.038	0.052	0.656
*P*-Quadratic	0.817	0.928	0.845	0.300
Weeks 12				
0 mg/kg FA	3.90	8.11	88.42	6.25
0.25 mg/kg FA	4.02	8.55	88.52	6.05
0.5 mg/kg FA	4.14	7.95	87.35	6.38
1 mg/kg FA	3.75	7.89	88.34	6.20
5 mg/kg FA	4.00	8.25	88.42	6.23
10 mg/kg FA	4.02	8.28	87.95	6.34
15 mg/kg FA	3.58	8.08	88.20	6.31
SEM	0.07	0.08	0.47	0.07
*P*-anova	0.399	0.439	0.936	0.997
*P*-linear	0.177	0.944	0.556	0.970
*P*-quadratic	0.202	0.583	0.899	0.996

**Table 4:** Effects of dietary supplementation with various levels of FA on egg 5-MTHF deposition of laying hens.

Item	5-MTHF, μg/100 g Dried Yolk			5-MTHF, μg/100 g Egg ^1^		
	4 Weeks	6 Weeks	12 Weeks	4 Weeks	6 Weeks	12 Weeks
0 mg/kg FA	222.98 ^e^	244.71 ^e^	286.42 ^c^	37.44 ^f^	41.28 ^f^	51.16 ^c^
0.25 mg/kg FA	286.78 ^e^	285.24 ^e^	310.69 ^c^	50.52 ^e^	49.42 ^ef^	55.19 ^c^
0.5 mg/kg FA	326.90 ^cd^	366.92 ^d^	358.50 ^c^	57.29 ^cd^	61.22 ^de^	63.01 ^c^
1 mg/kg FA	386.88 ^bc^	425.63 ^cd^	437.74 ^b^	68.45 ^bc^	73.28 ^cd^	77.61 ^b^
5 mg/kg FA	410.28 ^b^	477.10 ^bc^	511.73 ^a^	71.27 ^b^	82.58 ^bc^	90.05 ^ab^
10 mg/kg FA	542.57 ^a^	572.19 ^a^	520.20 ^a^	96.40 ^a^	100.29 ^a^	91.87 ^a^
15 mg/kg FA	566.92 ^a^	505.85 ^ab^	530.76 ^a^	98.93 ^a^	87.46 ^ab^	94.18 ^a^
SEM	20.52	19.78	17.35	3.64	3.52	3.05
*P*-ANOVA	<0.001	<0.001	<0.001	<0.001	<0.001	<0.001
*P*-Linear	<0.001	<0.001	<0.001	<0.001	<0.001	<0.001
*P*-Quadratic	0.031	<0.001	<0.001	0.022	<0.001	<0.001

^1^ 5-MTHF, μg/100 g egg: (5-MTHF content in egg yolk per egg/egg weight) × 100; ^a–f^ Different superscript notations within a single column indicate significant differences (*p* < 0.05).

**Table 5:** Effects of dietary supplementation with various levels of FA on egg FA deposition of laying hens.

Item	FA, μg/100 g Dried Yolk			FA, μg/100 g Egg ^1^		
	4 Weeks	6 Weeks	12 Weeks	4 Weeks	6 Weeks	12 Weeks
0 mg/kg FA	15.84 ^f^	15.73 ^e^	16.01 ^f^	2.68 ^f^	2.66 ^e^	2.85 ^f^
0.25 mg/kg FA	16.56 ^ef^	16.18 ^e^	16.73 ^f^	2.91 ^ef^	2.81 ^e^	2.98 ^ef^
0.5 mg/kg FA	18.62 ^de^	17.89 ^d^	18.12 ^e^	3.26 ^de^	2.99 ^e^	3.18 ^e^
1 mg/kg FA	19.44 ^d^	19.28 ^d^	19.51 ^d^	3.45 ^d^	3.32 ^d^	3.46 ^d^
5 mg/kg FA	22.13 ^c^	21.34 ^c^	22.51 ^c^	3.85 ^c^	3.69 ^c^	3.97 ^c^
10 mg/kg FA	25.99 ^b^	24.98 ^b^	24.17 ^b^	4.61 ^b^	4.38 ^b^	4.27 ^b^
15 mg/kg FA	29.93 ^a^	27.85 ^a^	27.69 ^a^	5.23 ^a^	4.82 ^a^	4.91 ^a^
SEM	0.79	0.69	0.64	0.14	0.13	0.11
*P*-ANOVA	<0.001	<0.001	<0.001	<0.001	<0.001	<0.001
*P*-Linear	<0.001	<0.001	<0.001	<0.001	<0.001	<0.001
*P*-Quadratic	0.240	0.036	0.021	0.173	0.027	0.040

^1^ FA, μg/100 g egg: (FA content in egg yolk per egg/egg weight) × 100; ^a–f^ Different superscript notations within a single column indicate significant differences (*p* < 0.05).

**Table 6:** Effects of dietary supplementation with various levels of FA on egg 5-FMTHF deposition of laying hens.

Item	5-FMTHF, μg/100 g Dried Yolk			5-FMTHF, μg/100 g Egg ^1^		
	4 Weeks	6 Weeks	12 Weeks	4 Weeks	6 Weeks	12 Weeks
0 mg/kg FA	10.85	7.37	7.06 ^d^	1.84	1.25	1.27 ^d^
0.25 mg/kg FA	11.74	10.92	10.99 ^ab^	2.07	1.88	1.96 ^ab^
0.5 mg/kg FA	8.56	8.38	11.86 ^a^	1.50	1.40	2.08 ^a^
1 mg/kg FA	8.58	9.72	8.37 ^cd^	1.52	1.68	1.49 ^cd^
5 mg/kg FA	10.89	9.31	9.32 ^cd^	1.89	1.61	1.64 ^bcd^
10 mg/kg FA	11.80	9.29	9.98 ^abc^	2.09	1.63	1.76 ^abc^
15 mg/kg FA	12.24	11.54	9.78 ^abc^	2.14	1.98	1.73 ^abc^
SEM	0.65	0.43	0.35	0.11	0.07	0.06
*P*-ANOVA	0.611	0.143	0.002	0.603	0.092	0.003
*P*-Linear	0.188	0.100	0.740	0.167	0.072	0.760
*P*-Quadratic	0.957	0.421	0.968	0.999	0.506	0.975

^1^ 5-FMTHF, μg/100 g egg: (5-FMTHF content in egg yolk per egg/egg weight) × 100; ^a–d^ Different superscript notations within a single column indicate significant differences (*p* < 0.05).

**Table 7:** Effects of dietary supplementation with various levels of FA on egg 10-FMF deposition of laying hens.

Item	10-FMF, μg/100 g Dried Yolk			10-FMF, μg/100 g Egg ^1^		
	4 Weeks	6 Weeks	12 Weeks	4 Weeks	6 Weeks	12 Weeks
0 mg/kg FA	38.44 ^e^	42.10 ^d^	44.05 ^c^	6.49 ^f^	7.12 ^d^	7.85 ^c^
0.25 mg/kg FA	44.74 ^e^	42.52 ^d^	53.66 ^bc^	7.87 ^ef^	7.38 ^d^	9.54 ^bc^
0.5 mg/kg FA	56.59 ^cd^	52.00 ^c^	58.43 ^ab^	9.90 ^cd^	8.68 ^cd^	10.25 ^ab^
1 mg/kg FA	52.08 ^d^	60.60 ^bc^	59.63 ^ab^	9.24 ^de^	10.46 ^b^	10.58 ^ab^
5 mg/kg FA	64.42 ^bc^	58.87 ^bc^	61.46 ^ab^	11.18 ^bc^	10.19 ^bc^	10.84 ^ab^
10 mg/kg FA	69.53 ^ab^	65.17 ^b^	66.03 ^a^	12.32 ^ab^	11.42 ^ab^	11.67 ^a^
15 mg/kg FA	76.21 ^a^	75.62 ^a^	67.03 ^a^	13.30 ^a^	13.04 ^a^	11.89 ^a^
SEM	2.21	2.12	1.71	0.39	0.38	0.30
*P*-ANOVA	<0.001	<0.001	0.002	<0.001	<0.001	0.002
*P*-Linear	<0.001	<0.001	<0.001	<0.001	<0.001	<0.001
*P*-Quadratic	0.030	0.500	0.156	0.020	0.358	0.183

^1^ 10-FMF, μg/100 g egg: (10-FMF content in egg yolk per egg/egg weight) × 100; ^a–f^ Different superscript notations within a single column indicate significant differences (*p* < 0.05).

**Table 8:** Effects of dietary supplementation with various levels of FA on egg total folate deposition of laying hens.

Item	Total Folate, μg/100 g Dried Yolk			Total Folate, μg/100 g Egg ^1^		
	4 Weeks	6 Weeks	12 Weeks	4 Weeks	6 Weeks	12 Weeks
0 mg/kg FA	288.12 ^e^	309.91 ^e^	353.55 ^d^	48.45 ^e^	52.30 ^e^	63.13 ^d^
0.25 mg/kg FA	359.81 ^de^	354.86 ^e^	392.07 ^cd^	63.37 ^d^	61.49 ^de^	69.67 ^cd^
0.5 mg/kg FA	410.66 ^cd^	445.18 ^d^	446.91 ^c^	71.95 ^cd^	74.29 ^d^	78.53 ^c^
1 mg/kg FA	466.98 ^bc^	515.23 ^cd^	525.25 ^b^	82.66 ^bc^	88.74 ^c^	93.14 ^b^
5 mg/kg FA	507.73 ^b^	566.62 ^bc^	605.03 ^a^	88.19 ^b^	98.07 ^bc^	106.50 ^a^
10 mg/kg FA	649.89 ^a^	671.63 ^a^	620.38 ^a^	115.42 ^a^	117.73 ^a^	109.58 ^a^
15 mg/kg FA	685.31 ^a^	620.85 ^ab^	635.25 ^a^	119.61 ^a^	107.30 ^ab^	112.71 ^a^
SEM	23.16	21.89	18.61	4.11	3.91	3.28
*P*-ANOVA	<0.001	<0.001	<0.001	<0.001	<0.001	<0.001
*P*-Linear	<0.001	<0.001	<0.001	<0.001	<0.001	<0.001
*P*-Quadratic	0.022	<0.001	<0.001	0.015	<0.001	<0.001

^1^ Total FA, μg/100 g egg: (Total FA content in egg yolk per egg/egg weight) × 100; ^a–e^ Different superscript notations within a single column indicate significant differences (*p* < 0.05).

## Data Availability

The data presented in this study are available on request from the corresponding author.
